# DNA G-quadruplex formation in response to remote downstream transcription activity: long-range sensing and signal transducing in DNA double helix

**DOI:** 10.1093/nar/gkt443

**Published:** 2013-05-28

**Authors:** Chao Zhang, Hong-he Liu, Ke-wei Zheng, Yu-hua Hao, Zheng Tan

**Affiliations:** State Key Laboratory of Biomembrane and Membrane Biotechnology, Institute of Zoology, Chinese Academy of Sciences, Beijing 100101, P. R. China

## Abstract

G-quadruplexes, four-stranded structures formed by Guanine-rich nucleic acids, are implicated in many physiological and pathological processes. G-quadruplex-forming sequences are abundant in genomic DNA, and G-quadruplexes have recently been shown to exist in the genome of mammalian cells. However, how G-quadruplexes are formed in the genomes remains largely unclear. Here, we show that G-quadruplex formation can be remotely induced by downstream transcription events that are thousands of base pairs away. The induced G-quadruplexes alter protein recognition and cause transcription termination at the local region. These results suggest that a G-quadruplex-forming sequence can serve as a sensor or receiver to sense remote DNA tracking activity in response to the propagation of mechanical torsion in a DNA double helix. We propose that the G-quadruplex formation may provide a mean for long-range sensing and communication between distal genomic locations to coordinate regulatory transactions in genomic DNA.

## INTRODUCTION

Transcription is controlled by proximal promoter and distal regulatory elements in genomic DNA in the upstream region of transcription start sites (TSS). Although a promoter may span around a TSS or locate proximately upstream to a TSS (1), regulatory elements, such as enhancers and silencers, are often thousands of base pairs (bps) away from TSS (2,3). To coordinate regulatory transactions, long-range communication among the transcription machinery and regulatory elements is required (4). For distal enhancers, investigation shows that the large spacer separating an enhancer and promoter can be ‘looped out’ to bring the elements into direct contact via associated proteins (5). It has also been proposed that enhancer complexes travel along the DNA until they meet the target promoter (6,7).

Although the aforementioned communication is directly mediated by proteins associated with the correspondent DNA elements, signals can also be transmitted through a DNA double helix itself. It has long been known that a moving polymerase generates negative and positive supercoilings behind and in front of the polymerase, respectively (8–10). Transcription-generated supercoiling can propagate over long distance in linear double-stranded DNA (dsDNA) *in vitro* (11) and in chromosomes *in vivo* (12). This supercoiling propagation dynamically melts the far upstream element of human *MYC* gene into single-stranded DNA and subsequently permits binding of the far upstream element-binding protein. These observations demonstrate that transcription induced mechanical stress can be transmitted to remote loci along a DNA double helix.

Besides the canonical dsDNA helix, guanine-rich DNA with four or more tandem guanine tracts can fold into a four-stranded secondary structure called G-quadruplex. G-quadruplexes have gained intense attention in recent years because of the prevalence of potential G-quadruplex sequences (PQS) in the genome and the implication of G-quadruplex structures in physiological and pathological processes, such as DNA replication, transcription and cancer development (13–15). PQS motifs are enriched in promoter regions, and G-quadruplexes are shown to serve as recognition elements that function through binding with regulatory proteins (16). The genome-wide probing using G-quadruplex ligand (17) and structural resolving by G-quadruplex specific proteins (18,19) provide strong support to the *in vivo* presence of G-quadruplex in eukaryotic genomes. Using engineered antibody, a recent work explicitly provides substantive evidence for the formation of G-quadruplex structures in the genome of mammalian cells (20). Given the prevalence of PQS in cells and their implication in physiological processes, how G-quadruplexes are generated in the genomes *in vivo* is an important aspect for understanding the physiological role of G-quadruplexes. Previously, experimental studies on G-quadruplex formation have mostly been carried out on single-stranded DNA in an isolated, static, rather than a physiologically functional environment. Because genomic DNAs, except the telomeric overhang, are present in double-stranded form and undergoes dynamic change in structure, information on G-quadruplex formation in a more physiologically relevant environment is desired.

In this work, we studied whether transcription-generated perturbation on dsDNA can induce G-quadruplex formation. In response to the two differential supercoiling states, G-quadruplex formation was observed in the region upstream, but not in the region downstream of a transcribed sequence. Interestingly, G-quadruplex formation can be efficiently induced at sites that are thousands of base pairs away from a promoter. The induction of G-quadruplex formation is insensitive to the distance over which a polymerase travels, but likely depends on and be proportional to the moving speed of the polymerase. The transcriptionally induced formation of G-quadruplex changes the recognition of the DNA by protein and causes transcription termination at the local region. In coincidence with the transcription orientation-dependence in G-quadruplex induction, genome sequence analysis revealed a preferential enrichment of PQS motifs in the region upstream, but not in the region downstream of the transcribed region of genes in warm-blooded animals. This biased enrichment of PQS motifs supports a biological function of G-quadruplex induction in transcription. Collectively, our results demonstrate that the PQS motifs can sense remote DNA tracking activity in response to the propagation of mechanical disturbance on DNA structure and, in turn, regulate local physiological activities.

## MATERIALS AND METHODS

### Preparation of dsDNA

**Figure 1.**
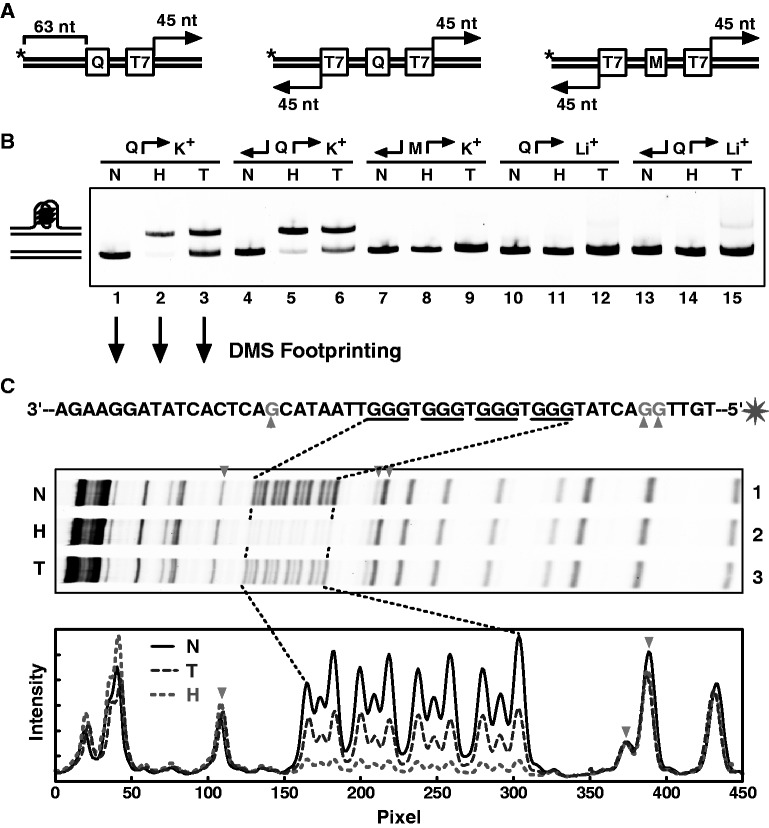
G-quadruplex formation induced by downstream transcription. (**A**) Schemes illustrating the structure of the DNAs in which a G_3_(TG_3_)_3_ motif (Q) or its mutant (M) was placed upstream of one or between two divergently oriented T7 promoters (T7), which was flanked by 45 bp at its 3′ side so that transcription can proceed for 45 nucleotides (nt). (**B**) G-quadruplex formation in transcribed DNA detected by native gel electrophoresis. DNAs with a FAM dye at the 5′-end of the nontemplate strand were subjected to transcription followed by digestion with RNase A and H or heat denaturation/renaturation. The structure of the DNAs and cation in the transcription buffer is indicated above the gel. N, H, T indicates non-transcribed, heated (21,22) and transcribed DNA, respectively. The schemes at the left illustrate the structures of the corresponding DNA bands. (**C**) Verification of G-quadruplex formation by DMS footprinting. Cleavage fragments were resolved on a denaturing gel (top) and digitized (bottom) for comparison.

**Figure 2.**
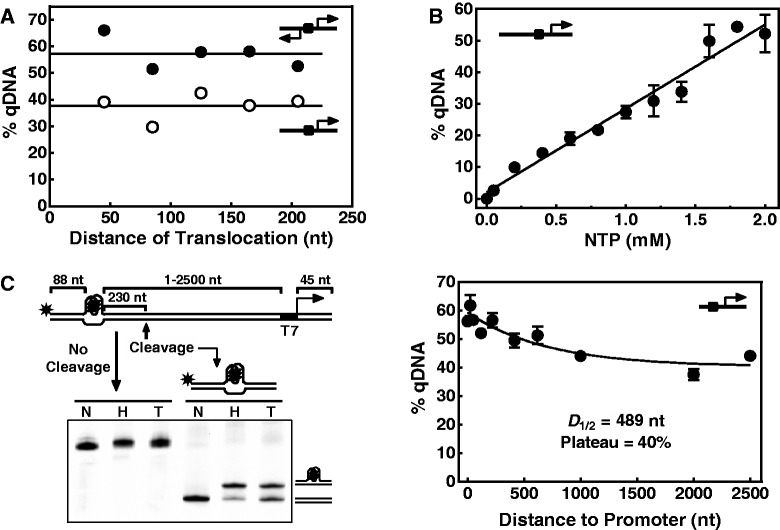
Characterization of G-quadruplex formation induced by downstream transcription. DNA carrying a G_3_(TG_3_)_3_ was transcribed with T7 polymerase and resolved on a native gel. G-quadruplex-bearing DNA was expressed as percentage of total DNA (% qDNA). (**A**) G-quadruplex formation as a function of the distance a polymerase could translocate. One group of DNAs carried a G_3_(TG_3_)_3_ between two divergently oriented T7 promoters (filled circle). Another group of DNAs was derived from this group by mutating the T7 promoter in the upstream region (open circle). The size of the sequences flanking the 3′ side of the T7 promoter(s) varied from 45–205 bp. (**B**) G-quadruplex formation as a function of NTP concentration. DNA bearing a G_3_(TG_3_)_3_ upstream of a T7 promoter (Figure 1A, scheme at left) was transcribed at various concentrations of NTP and resolved on a native gel. Data show the mean and standard deviation of three independent experiments. (**C**) G-quadruplex formation in long dsDNA. The DNAs contained a G_3_(TG_3_)_3_ upstream of a T7 promoter, which were separated by various length of base pairs (left panel). DNA of 600 bps or longer was cut at a restriction site 230 bp downstream of the G_3_(TG_3_)_3_ after transcription, producing a FAM-labeled 333-bp fragment that could be resolved by native gel electrophoresis. The remaining samples of shorter DNA were treated under the same condition, but in the absence of restriction enzyme. Data (right panel) show the mean and standard deviation of three independent experiments. *D*_1/2_ denotes the distance required for G-quadruplex formation to drop to the midvalue between the minimum and maximum.

The dsDNAs used for Figures 1, 2A (the shortest) and B, Supplementary Figures S2 and S3 and the three short dsDNAs for Figure 2C were prepared by overlap PCR as described (21). To prepare the other dsDNAs for Figure 2A, a synthetic DNA fragment containing a G_3_(TG_3_)_3_ motif upstream of a T7 promoter or between two divergently oriented T7 promoters was inserted into the TA cloning site of pMD-19-T simple plasmid (TaKaRa Biotech, Dalian, China). The construct was then transformed into *Escherichia coli* JM109 (TransGen Biotech, China), and the resulting plasmid was purified and used as template to obtain the dsDNAs of different sizes spanning the insert by PCR (Supplementary Figure S1A). To prepare the dsDNAs of 120 bp or longer for Figure 2C, a synthetic DNA fragment containing a G_3_(TG_3_)_3_ motif upstream of a T7 promoter was inserted into the TA cloning site of pMD-19-T simple plasmid. Then, dsDNAs of different sizes were inserted at the EcoR I/Hind III site between the G_3_(TG_3_)_3_ motif and T7 promoter to expand the distance between them. The construct was transformed into *E.coli* JM109, and the resulting plasmid was purified and used as template to obtain the dsDNAs in which the G_3_(TG_3_)_3_ was separated by various base pairs from the T7 promoter by PCR (Supplementary Figure S1B). All PCR used a 5′-FAM labeled upstream primer.

### In vitro transcription

Transcription was carried out essentially as described (21) with minor modifications. One pmole dsDNA was incubate at 37°C for 1 h in a 25 µl of mixture of 40 mM Tris–HCl (pH 7.9), 8 mM MgCl_2_, 10 mM dithiothreitol (DTT), 2 mM spermidine, 50 mM KCl or LiCl, 40% (w/v) polyethylene glycol (PEG) 200, 20 U T7 RNA Polymerase (Fermentas, Thermo scientific), 2 mM nucleoside triphosphates (NTPs), 0.15 U inorganic pyrophosphatase (Fermentas, Thermo scientific). Transcription with T3 and SP6 polymerase was carried out as aforementioned, but scaled up to 100 µl volume.

### Detection of G-quadruplex formation

Transcribed DNA was mixed with equal volume of solution containing 10 µg of RNase A (Fermentas, Thermo scientific), 5 U RNase H (Fermentas, USA), 50 mM KCl or LiCl, 40% (w/v) PEG 200 and incubated at 37°C for 1 h. The digestion reaction was stopped by addition of 2 µl of 0.5 M EDTA, followed by an 1 h digestion at 37°C with 40 µg of Proteinase K (TaKaRa Biotech, China). For the long DNAs used in Figure 2C, 3 µl of RNase A and H treated sample was incubated with the FastDigest Kpn 21 (Fermentas, USA) at 37°C for 15 min in a total volume of 25 µl containing 2 µl 10× FastDigest Buffer, 50 mM KCl, 1 µl FastDigest Kpn 21. Reaction was terminated by addition of 0.1% SDS (final concentration), followed by incubation for 3 min at room temperature. G-quadruplex formation in the DNA was detected by native gel electrophoresis as described (21). In some experiments, DNA was incubated with 375 nM of nucleolin for 30 min at 4°C before electrophoresis.

### Computational analysis of PQS in genomes

Sequences of protein-coding genes were downloaded in fasta format from the Ensembl genes database with the BioMart tool (http://www.ensembl.org/). We used a home-written Perl script to find PQS motifs and their coordinates using a pattern matching code G{3,}(.{1,7}?G{3,}){3,} that represents a DNA sequence G_≥3_(N_1__–__7 _G_≥3_)_≥3_, where G denoted guanine and N denoted any nucleotide, including G. To obtain the combined cumulative distribution of PQS-positive genes upstream of TSS, a list of PQS motifs with their gene IDs and coordinates was collected from both the non-template and template DNA strands using the aforementioned algorithm. The list was sorted by coordinates in a descending order and then fed into a Perl hash where the gene IDs was stored as hash keys. During this process, the number of PQS motifs and PQS positive genes accumulated to a specific coordinate was obtained. The Perl source codes and their correspondent compiled standalone executable files are provided in the Supplementary Data (Supplementary package).

## RESULTS

### Transcription induces G-quadruplex formation in upstream region

Transcription along a DNA double helix generates negative supercoiling behind a moving polymerase and positive supercoiling in front of it (9,23). To inspect how a negative supercoiling would affect G-quadruplex formation, we constructed dsDNAs containing a PQS motif 5′-G_3_(TG_3_)_3_-3′ or a mutant 5′-GCGTGGCTCCGTCGC-3′, upstream of one or two divergently oriented T7 promoters (Figure 1A). This motif was chosen for its relatively high stability to facilitate structure detection. To examine the physiological relevance of such motifs, bioinformatic searching found >800 PQS motifs with G_3_ tracts and 1-nt loops within the 5000-nt region immediately upstream of the TSS in 22 049 human protein-coding genes. Transcription was initiated by supplying T7 polymerase and the four NTPs in a solution containing 40% PEG 200 and 50 mM of K^+^ or Li^+^. PEG was added to stabilize G-quadruplex formation in dsDNA to facilitate its detection (21). After transcription was terminated, the RNA transcripts were digested with RNase A and H. A post-transcription digestion with RNase results in clean DNA bands (22). A separate DNA sample was also incubated along with the transcribed sample under the same temperature at the same time. Formation of intramolecular G-quadruplex in the DNA was then detected by native gel electrophoresis in which the DNA bearing a G-quadruplex migrated behind the same DNA without G-quadruplex. As a positive control, a same DNA sample was subjected to heat denaturation/renaturation to generate G-quadruplex without transcription (21).

G-quadruplex formation was detected in the two transcribed dsDNAs carrying the G_3_(TG_3_)_3_ motif. This was indicated by the appearance of an additional slower-migrating band (Figure 1B, lanes 3 and 6) at the same position as the G-quadruplex-bearing DNA band in the heated samples (lanes 2 and 5). The formation of G-quadruplexes was specifically induced by the transcription because the DNAs that were subjected to the same incubation processes but without transcription did not form secondary structure (Figure 1B, lanes 1 and 4; Supplementary Figure S2). Transcription with two promoters oriented in divergent directions intensifies negative supercoiling. As a result, the amount of G-quadruplex in the DNA carrying two promoters was apparently more than that in the DNA carrying just one promoter (Figure 1B, lane 6 versus 3). This result is in agreement with the report that superhelicity in dsDNA facilitates spontaneous G-quadruplex formation in plasmid (24). The G-quadruplex formation was specific to the G_3_(TG_3_)_3_ motif because the DNA carrying a mutated motif did not show extra band (Figure 1B, lanes 8 and 9). Because Li^+^ does not stabilize G-quadruplex (25), G-quadruplex formation was almost invisible when the heat denaturation/renaturation (Figure 1B, lanes 11, 14) or transcription (Figure 1B, lanes 12, 15) was conducted in Li^+^ instead of in K^+^ solution.

To verify the G-quadruplex structure in the heated and transcribed DNAs (Figure 1B, lanes 2 and 3), dimethyl sulfate (DMS) footprinting was performed (Figure 1C). Guanine residuals in a G-quadruplex are protected from methylation by DMS at the N7 and subsequent chemical cleavage (26). As is shown in Figure 1C, the four guanine tracts were all protected in the heated and transcribed DNA samples. Collectively, the aforementioned results indicated that the negative supercoiling generated by transcription induced G-quadruplex formation in the dsDNAs upstream of the TSS.

### G-quadruplex formation is insensitive to the distance a polymerase travels

To characterize transcription-induced G-quadruplex formation, we next examined how the distance that a polymerase translocates would affect the formation of G-quadruplex. In these experiments, the PQS motif was placed at a fixed position upstream of a T7 promoter and sequences downstream of the promoter varied in size so that a polymerase could travel for different distances. G-quadruplex formation in the transcribed DNAs was quantitated by native gel electrophoresis and expressed as percentage of the total DNA transcribed. The results in Figure 2A show that G-quadruplex formation is insensitive to the distance the polymerase could travel. In agreement with the result in Figure 1B (lane 6 versus 3), arranging a promoter on both sides of the PQS motif enhanced G-quadruplex formation. However, its magnitude remained constant, regardless of the translocation distance of the polymerase.

### G-quadruplex formation is proportional to NTP concentration

We further investigated the dependence of G-quadruplex formation on NTP concentration. Transcriptions were carried out at various NTP concentrations, and G-quadruplex formation was quantitated by native gel electrophoresis. In Figure 2B, the data show that the amount of G-quadruplex-bearing DNA increased with an increase in NTP concentration. From a biochemical perspective, NTP concentration determines how fast a polymerase can obtain a building block, i.e. a NTP, from the medium before the RNA synthesis proceeds to the next nucleotide on the template. Previous studies showed that the rate of polymerase translocation on DNA is positively proportional to NTP concentration (27,28). Negative and positive supercoiling are generated by the movement of a polymerase. We infer that faster translocation builds up greater torsion and, as such, enhances the G-quadruplex formation.

### Long-distance induction of G-quadruplex formation by transcription

The abnormal supercoiling generated by a moving polymerase propagates along a linear dsDNA (29). Without topological constraint, the DNA would rotate at the same time to relax the tension to restore the native helicity of the DNA helix (30). This is expected to result in decay in the degree of torsion, as it propagates away from where it was generated. To explore how this relaxation would affect the G-quadruplex formation, we made DNAs in which the PQS motif was placed at various distances upstream to the T7 promoter that was flanked by a 45 bp sequence at its downstream 3′ side. The result in Figure 2C (right panel) shows that G-quadruplex formation was more effective when the PQS motif was placed closer to the promoter, but it decayed slowly as the distance between the promoter and PQS motif increased. G-quadruplex formation occurred even when the PQS motif was placed 2500 bp apart from the promoter. Fitting the data points to a single-phase exponential decay function, Y = max*exp(-k*X) + plateau, resulted in max = 59%, plateau = 40%, with a half decay distance (*D*_1/2_) of 489 bp. The slow decay and a significantly large plateau in G-quadruplex formation imply that the propagation of the supercoiling is a faster process that passes through a DNA far before meaningful relaxation in DNA torsion takes place. This interpretation is in agreement with the results in two single-molecular transcription studies. A study using *E.coli* RNA polymerase demonstrated that a 4971 bp dsDNA rotates at a rate of ∼1 revolution per second at optimal NTP concentration (30), whereas the other revealed that a structural reorganization of DNA into plectoneme driven by DNA twisting could propagate over long-distance on a millisecond timescale (31).

### Transcription-generated positive supercoiling does not induce G-quadruplex formation

Transcription creates positive supercoiling in front of a moving polymerase (9,23). To find out whether positive supercoiling would induce G-quadruplex formation, a PQS motif was placed downstream of the T7 promoter. Transcription was carried out in the presence of only three NTPs so that the translocation of a polymerase would stall at the first adenine upstream of the PQS motif because of the lack of UTP. The results in Supplementary Figure S2 show that no G-quadruplex could be detected. It can be imagined that a DNA helix would be twisted further with the generation of positive supercoiling, resulting in an overwound state. Therefore, the positively supercoiled state is likely inhibitive to G-quadruplex formation.

### Preferential selection of G-quadruplex sequences upstream of TSS

**Figure 3.**
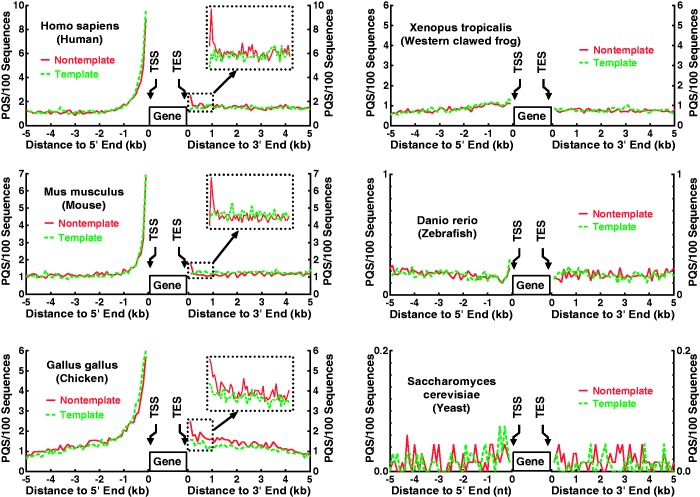
Distribution of PQS occurrence in the 5000 bp region flanking the 5′ and 3′ end of genes. Frequency was normalized to the number of sequences and expressed as the number of occurrences in 100 sequences within a 100 nt window. A gene here denotes the transcribed region, including 5′ untranslated region (UTR), exons, introns and 3′ UTR. TSS and TES indicate transcription start and end site, respectively. Inserts in the left panels show PQS mapping at 20 nt resolution within the 1000 nt region downstream of TES.

Our results show that the induction of G-quadruplex formation is transcription orientation-dependent. In a physiological context, it implies G-quadruplex forms in the upstream region of the transcribed sequence of a gene and is suppressed in the downstream region of a gene. To explore the physiological reality of this polarized G-quadruplex formation, we searched for PQS in the genome of human and other species in the 5000 bp regions flanking the 5′ and 3′ end of protein-coding genes, respectively. Interestingly, polarized distribution of PQS occurrence was obtained in the warm-blooded animals (Figure 3), which correlates with the orientation dependence of G-quadruplex formation driven by transcription. In the upstream regions, there is a significant enrichment of PQS far above the background, which is in agreement with several previous works showing enrichment of PQS motifs in the vicinity around TSS (32–35). The peak value of the frequency of PQS occurrence in human and mouse is five times higher than the background value. However, there is little enrichment in the downstream regions. This fact strongly suggests that the PQS have been positively selected during natural evolution. In contrast to the warm-blooded animals, polarized enrichment of PQS was not found for zebrafish and fruitfly. These results suggest that a systematic selection of G-quadruplexes as regulatory elements occurred in the warm-blooded animals as others (33) and we reported (22). In lower organisms, G-quadruplex may work in individual cases. In a previous study, a biased enrichment of PQS in the non-template verses template strand within the 100 nt region 3′ of transcription end site (TES) has been found in human genome (36). We also detected this feature in human, mouse and chicken (Figure 3, inserts in left panels). However, the magnitude of the enrichment is still far less than those upstream of TSS.

**Figure 4.**
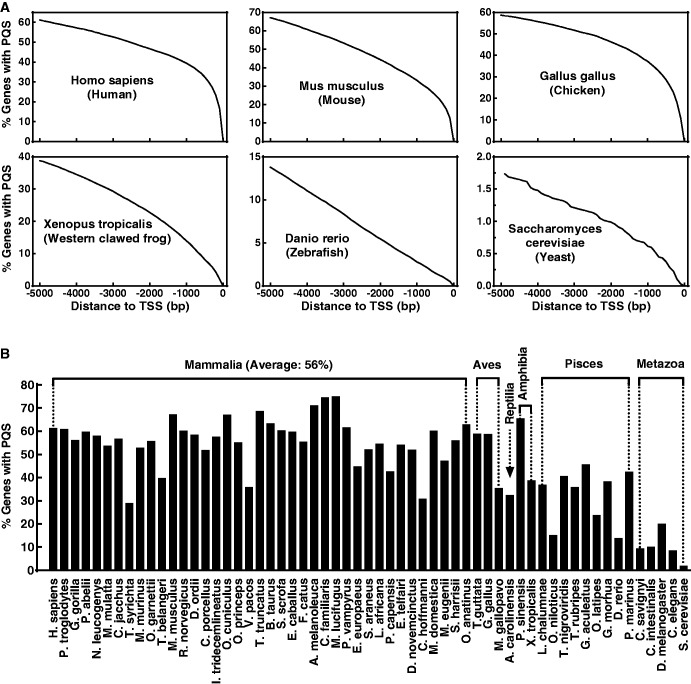
Statistics of genes carrying PQS motifs in different species. (**A**) Percentage genes having PQS in the upstream region of their TSS. The value at a given position gives the percentage of genes that have at least one PQS in the range from −1 to the given position. (**B**) Percentage genes having PQS within the −1 to −5000 bp upstream region of their TSS. The species is ordered according to the species tree provided on the Ensembl website. PQS on both the template and non-template strand was counted in (A) and (B).

In Figure 4A, the probability of finding a PQS in the upstream region of the TSS of genes in six species is given. It shows that ∼60% of the genes in human, mouse and chicken have at least one PQS within the 5 kb region upstream of their TSS. However, the percentage of PQS-positive genes in lower species is dramatically smaller, being ∼40% in frog, <15% in zebrafish and ∼1.75% in yeast. To obtain an overview of the G-quadruplex-forming sequences present in different species, we carried out a genome-wide searching for the 69 species available in the Ensembl database (Figure 4B). It is seen that >50% of genes in mammalians have PQS within the 5 kb region upstream of their TSS in average. Although the number of species in the aves, reptilia and amphibia is too small for a general conclusion, the percentage of PQS-positive genes in the pisces and metazoa is much lower.

## DISCUSSION

**Figure 5.**
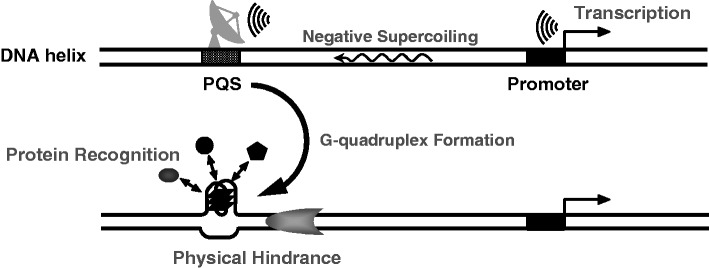
Model of downstream transcription activity sensing by G-quadruplex formation and its direct effect on DNA. The fast propagation of negative supercoiling generated by a proximal or distal downstream transcription or DNA tracking event induces a G-quadruplex formation at the PQS site and subsequently affects protein recognition and hinders protein translocation along the DNA.

Recently, we described a co-transcriptional formation of DNA:RNA hybrid G-quadruplex structures that occurs downstream of TSS (22). In the current work, we further demonstrated that how G-quadruplex formation can be induced upstream of TSS by transcription. Together, these two works illustrate how G-quadruplex structures can be induced in dsDNA in a dynamic physiological process. In the present study, our bioinformatic analysis suggests that the biased induction of G-quadruplex formation with respect to the upstream and downstream region of transcription is evolutionally selected in warm-blooded animals. The experimental data show that G-quadruplex formation in dsDNA can be triggered by a transcription event that is thousands of base pairs away. This feature may provide a mechanism to monitor or sense remote transcription activities (Figure 5). Similar to the other structural alterations, like the strand melting,(11,12), the sensing by PQS is real-time, remote (Figure 2C, right panel) and measures the transcription activity (Figure 2B). In a linear DNA, transcriptionally generated torsion dissipates with time because the DNA is free to rotate. Under this condition, G-quadruplex formation is still effectively induced in locus far away from where transcription takes place. This fact implies that the rate of torsion propagation is much faster than the rate at which the torsion dissipates, thus ensures a real-time long-range signal transmission.

Unlike strand melting (11,12) and plectoneme (31) formation that are largely sequence-independent and advance along with the propagation of supercoiling, G-quadruplex formation is fixed at a specific site where PQS locates. This may be useful when a regulation at a specific locus is required. G-quadruplex formation alters the structure of a DNA helix and is know to affect local activities in two aspects. First, it influences recognition of regulatory proteins (13). This functionality is first illustrated by the early work of the Hurley group who demonstrated that an intramolecular G-quadruplex structure upstream of the P1 promoter of C-MYC controls the transcriptional activation of the gene (37). Another example is that hnRNP K and nucleolin have been shown to act as transcriptional activators of the VEGF promoter through interaction with G-quadruplex (38). Second, G-quadruplexes have been shown to hinder translocation of helicase (39) and cause transcription arrest (40) when located in front of a moving protein. Therefore, G-quadruplex also acts as a physical hindrance that impairs the translocation of motor proteins on a DNA tract. This may influence a number of essential physiological processes like transcription, DNA replication and repair. In agreement with the previous findings, our supplementary data show that co-transcriptional formation of G-quadruplex affected the interaction of nucleolin to two dsDNAs carrying G-quadruplex-forming sequences from the C-MYC and VEGF gene (Supplementary Figure S3A), respectively, and caused transcription termination (Supplementary Figure S3B). For the aforementioned reasons, we infer that the induction of G-quadruplex formation offers a mean to regulate local activity in response to a remote downstream transcription event.

Negative supercoiling is generated by DNA-tracking activities of motor proteins such that it occurs in a number of cellular processes, like DNA replication and repair, in addition to transcription. Therefore, we speculate that the remote induction of G-quadruplex formation may also occur in such cellular processes. A G-quadruplex-forming motif, in general, may function as a sensor or receiver that response to propagation of tortional stress so that it monitors DNA-tracking activity at downstream regions (Figure 5). Because of the long-range capability in G-quadruplex induction, this can establishes a communication between distal genomic locations to coordinate regulatory transactions in genomic DNA. In response to downstream tracking activity, a G-quadruplex formed can regulate local activity by altering the recognition of relevant proteins to their binding sites near a PQS and impairing the movement of motor proteins on the DNA. Given the abundance of PQS in genomes, G-quadruplex formation in response to the propagation of abnormal supercoiling generated by transcription or other DNA tracking activities in genomic DNA may have important implications for DNA-dependent physiological processes.

## SUPPLEMENTARY DATA

Supplementary Data are available at NAR Online: Supplementary Package and Supplementary Figures 1–3.

## FUNDING

[2013CB530802], [2012CB720601] and [2010CB945300] from the Ministry of Science and Technology of China; [30970617] and [21072189] from the National Science Foundation of China. Funding for open access charge: Ministry of Science and Technology of China [2012CB720601].

*Conflict of interest statement.* None declared.

## Supplementary Material

Supplementary Data
