# Supplementary material for: DNA G-quadruplex formation in response to remote downstream transcription activity: long-range sensing and signal transducing in DNA double helix
Source: Nucleic Acids Res. 2013 May 28;41(14):7144–52. doi: 10.1093/nar/gkt443 (PMC3737545; doi:10.1093/nar/gkt443)
Supplement: Supplementary Data [file supp_41_14_7144__index.html]

DNA G-quadruplex formation in response to remote downstream transcription activity: long-range sensing and signal transducing in DNA double helix — DNA G-quadruplex formation in response to remote downstream transcription activity: long-range sensing and signal transducing in DNA double helix — Supplementary Data 

# DNA G-quadruplex formation in response to remote downstream transcription activity: long-range sensing and signal transducing in DNA double helix

## Supplementary Data

files

**Files in this Data Supplement:**

- Supplementary Data - pdf file
