# Supplementary material for: DNA G-quadruplex formation in response to remote downstream transcription activity: long-range sensing and signal transducing in DNA double helix
Source: Nucleic Acids Res. 2013 May 28;41(14):7144–52. doi: 10.1093/nar/gkt443 (PMC3737545; doi:10.1093/nar/gkt443)
Supplement: Supplementary Data [file supp_gkt443_nar-00945-f-2013-File003.pdf]

Supplementary Data for

DNA G-quadruplex formation in response to remote downstream transcription activity: Long-range sensing and signal transducing in DNA double helix

Chao Zhang, Hong-he Liu, Ke-wei Zheng, Yu-hua Hao, Zheng Tan*

State Key Laboratory of Biomembrane and Membrane Biotechnology, Institute of Zoology, Chinese Academy of Sciences, Beijing 100101, P. R. China

*E-mail: z.tan@ioz.ac.cn; tanclswu@public.wh.hb.cn

1 supplementary package and 3 supplementary figures

Supplementary package (scripts, compiled stand-alone executable files and sample files)

The Perl source codes were developed using the Active Perl 5.14.2 (downloaded from www.activestate.com/activeperl) under the Windows OS. Extract the files in the package into a folder using the 7-Zip free software (www.7-zip.org). Place sequence files in the same folder. Double click the transcript or standalone executive file will analyze all the sequence files in the folder. Please see instructions in the package.

Supplementary_package.7z

Supplementary Figure S1

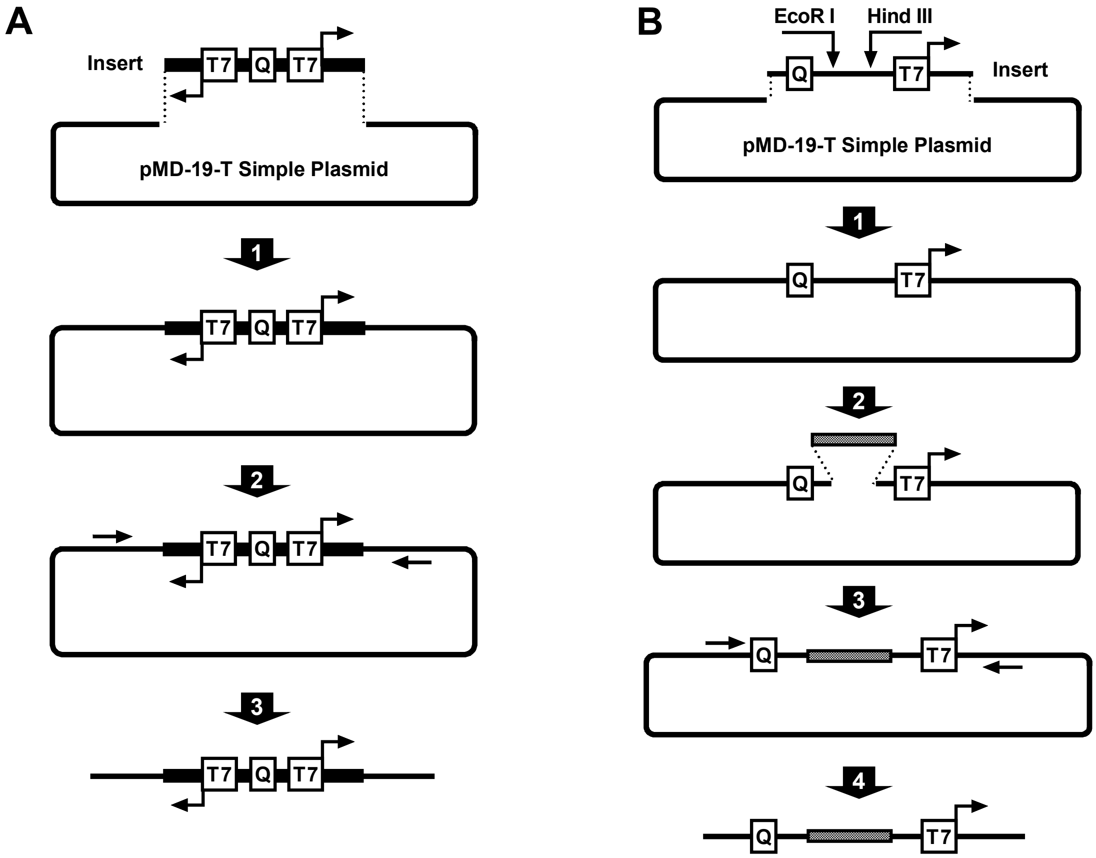

Fig. S1. (A) Preparation of dsDNA in which the T7 promoter is flanked at its 3' side with sequences of various sizes. A fragment carrying a PQS motif and T7 promoter was inserted into a plasmid. Then a region spanning the PQS motif and T7 promoter in the resulting plasmid was amplified by PCR. The size of the sequences flanking the 3' side of the T7 promoter was adjusted by moving the two primers close or apart. (B) Preparation of dsDNA in which the PQS motif was separated from the T7 promoter by various distances. A fragment carrying a PQS motif and T7 promoter as well as two restriction sites between them was inserted into a plasmid. Then fragments of different sizes were inserted into the restriction sites to obtain the desired distance between the PQS motif and T7 promoter. The region spanning the PQS motif and T7 promoter in the resulting plasmid was amplified by PCR. The size of the amplicon was adjusted by moving the two primers (straight arrows) close or apart.

Supplementary Figure S2

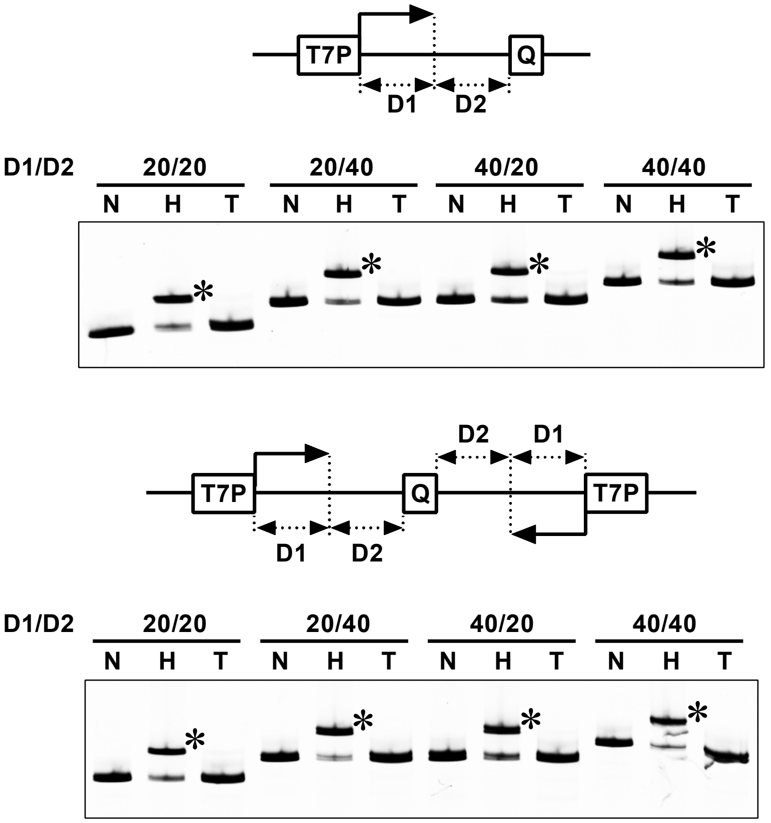

Fig. S2. Transcription did not induce G-quadruplex formation downstream of transcribed sequence. DNAs bearing a $G_3(TG_3)_3$ motif (Q) were transcribed with one or two T7 promoters (T7P) arranged as indicated and resolved on a native gel. N, H, T indicates non-transcribed, heated and transcribed DNA, respectively. Asterisk indicates DNA bearing a G-quadruplex generated by heat denaturation/renaturation.

Supplementary Figure S3

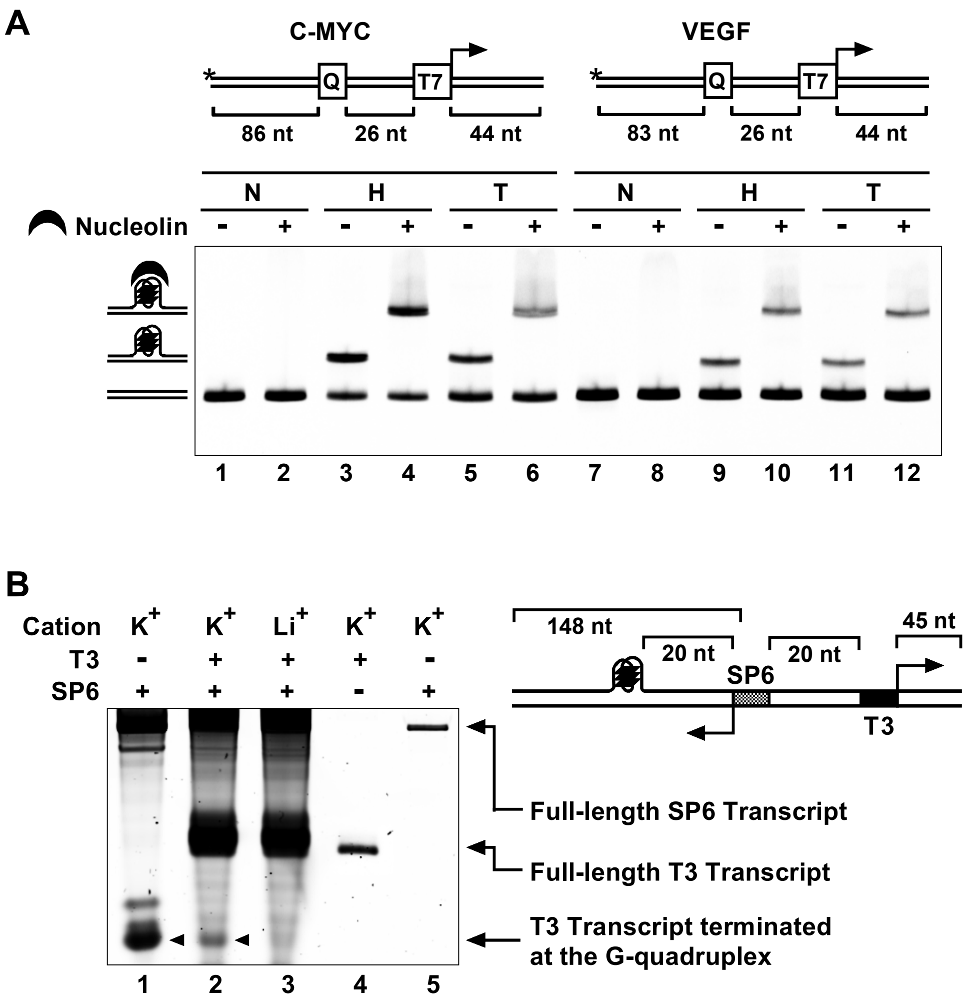

Fig. 3. Effect of G-quadruplex formation induced by transcription. (A) Binding of nucleolin to PQS target. DNA carried a PQS motif from the C-MYC (5'-AGGGTGGGGAGGGTGGGGAA-3') or VEGF (5'-CGGGGCGGGCCGGGGGCGGGGT-3') gene. DNA without transcription (N), heated (H), or transcribed (T) was incubated with nucleolin before electrophoresis. The binding of nucleolin caused further migration retardation to G-quadruplex-bearing DNA due to the formation of protein-DNA complex. Schemes at the left illustrate the structures of the correspondent DNA bands. Nucleolin is a multifunctional phosphoprotein that has been reported to bind, under both *in vitro* and *in vivo* conditions, to the G-quadruplex in the proximal promoter of C-MYC. By stabilizing C-MYC G-quadruplex, nucleolin suppresses the activity of the C-MYC promoter (1). Nucleolin also binds selectively to the G-quadruplex in the VEGF promoter

and positively regulates its transcription (2). (B) G-quadruplex formation induced by T3 polymerase caused transcription termination to SP6 polymerase. A DNA, carrying a $(G_3T)_3G_3$ and divergently oriented T3 and SP6 promoters, was first transcribed with T3 polymerase for 0.5 hr and then SP6 polymerase for 1 hr. The RNA transcripts were resolved on a denaturing gel and visualized by staining with SYBR Gold dye. Because staining intensity is proportional to the size of RNA and the RNA terminated at the G-quadruplex (arrowhead) was 7-8 times shorter than the SP6 full-length RNA, the termination efficiency is far more effective than it appeared on the gel. The DNA in lane 1 was heated to generate G-quadruplex before transcription with SP6 only.

Expression of nucleolin

A DNA fragment was obtained from nucleolin mRNA by RT-PCR using a primer pair of ACTGAGCTAGCATGGCCAAACAGAAAGCA and TATGTCTCGAGTTAAAACTTCGTC-TTCTTTCCTTG. The fragment was inserted into the Nhe I/Xho I site of a pET-28b plasmid (Novagen, USA), then transformed into E. coli strain BL21 (DE3). Nucleolin expression was induced with 1 mM isopropyl thiogalactoside (IPTG) and the N-his tagged nucleolin was purified with the His GraviTrap column (GE Healthcare, USA).

Analysis of transcription termination

Transcription sample was extracted with phenol/chloroform/isoamylol (25:24:1). The RNA in the aqueous phase was precipitated with ethanol, resuspended in formamide dye, and denatured at 95 °C for 3 min. After being resolved on a 10% denaturing polyacrylamide gel containing 7 M urea, 90 mM Tris-borate, 2 mM EDTA, the RNA was stained with SYBR Gold (Life technologies, USA). The gels were scanned on a Typhoon 9400 phosphorimager (GE Healthcare, USA).

References

1. Gonzalez, V., Guo, K., Hurley, L. and Sun, D. (2009) Identification and characterization of nucleolin as a c-myc G-quadruplex-binding protein. *J. Biol. Chem.*, **284**, 23622-23635.
2. Uribe, D.J., Guo, K., Shin, Y.J. and Sun, D. (2011) Heterogeneous nuclear ribonucleoprotein K and nucleolin as transcriptional activators of the vascular endothelial growth factor promoter through interaction with secondary DNA structures. *Biochemistry*, **50**, 3796-3806.
